# The Effects of Occupational Status and Sex-Typed Jobs on the Evaluation of Men and Women

**DOI:** 10.3389/fpsyg.2018.01170

**Published:** 2018-07-12

**Authors:** Cristina García-Ael, Isabel Cuadrado, Fernando Molero

**Affiliations:** ^1^Department of Social and Organizational Psychology, Faculty of Psychology, Universidad Nacional de Educación a Distancia (UNED), Madrid, Spain; ^2^Area of Social Psychology, Faculty of Psychology, University of Almería, Almería, Spain

**Keywords:** status-competition, competence-warmth, emotions, behavioral tendencies, gender

## Abstract

**Background:** Occupational segregation by gender is one of the major problems faced by professional women in the labor market. Since the sixties, psychological explanations point out that gender stereotypes are responsible for this persistent inequality in the workforce. Nevertheless, most of research has overlooked that emotions are particularly important as the discrimination faced by professional women is better explained by the ambivalent feelings they provoke than by stereotyping.

**Aim:** The aim of this research is to analyse from the Stereotype Content Model (SCM, Fiske et al., 2002) and the Behaviors from Intergroup Affect and Stereotypes (BIAS) Map (Cuddy et al., 2007) whether cognitive, affective and behavioral components of prejudice act jointly to explain gender segregation in the labor market.

**Method:** 1098 Spanish workers (59% women) from different occupational sectors were requested to rate how professional men and women in high (leaders) and low status (secretaries) positions who work in male (high-tech company) and female-dominated (health company) occupations are perceived (stereotypes), as well as the affective responses and the behavioral tendencies that they arouse.

**Data analyses:** Two analyses of variance (a) and two ANOVAs with repeated measures (b) were carried out to analyze the effect of occupational status (high vs. low), type of company (high-tech vs. health) and workers’ sex (men vs. women) on: (a) the social structural variables (status and competition), (b) on the stereotyped dimensions (competence and warmth) and (c) on emotions (admiration, envy and contempt). Finally, mediational analyses were carried out to examine the link between stereotypes, emotions, and behavioral tendencies.

**Results:** The most striking results show that (a) competition and status differentiate leaders and secretaries, (b) men leaders are rated as more competent and less warm than secretaries, whereas women leaders are viewed as more competent than women secretaries but with equivalent warmth, and (c) admiration and envy predict behavioral tendencies, but restricted to professional men regardless of organizational context.

**Conclusion:** Results reveal that cognitive, affective and behavioral components of prejudice act jointly to explain discrimination against women in the workplace. Findings are discussed according to the SCM and the BIAS Map.

## Introduction

During the past 50 years, women have become incorporated into the formal labor market in vast numbers. However, there can be no talk of full integration because of the gender-related occupational segregation which characterizes the job market (Huffman et al., 2010). Gender segregation in the workforce has both vertical and horizontal aspects (Hakim, 1993). The horizontal aspect (occupational segregation) concerns the different types of work that men and women perform, and the vertical aspect (occupational inequality) refers to the hierarchical disparities in their work (e.g., Baunach, 2002). Both are intricately connected and allow that women are overrepresented in female-dominated occupations, and men in male-dominated occupations.

Since the sixties, psychosocial research has demonstrated the impact of stereotypes (consensual beliefs about typical traits of women and men) on judgments about professional men and women (Broverman et al., 1972). Specifically, many scholars argue that the typical characteristics of women (feminine) and men (masculine), as well as their traditional roles (men as providers vs. women as homemakers) spill over into the workplace, leading to discrimination against women and to a gender-segregated labor market (Eagly, 1987). However, it must be highlighted that research into this subject has been almost always focused on stereotypes (cognitive aspects of prejudice) (Bosak et al., 2012). In our opinion, the affective dimension is likely to be particularly important for professional women, mainly because the discrimination to which women are exposed in the workforce can best be explained by ambivalent feelings (Wade and Brewer, 2006). Therefore, the main aim of this research is to examine from the Social Role Theory (SRT; Eagly, 1987), the Stereotype Content Model (SCM; Fiske et al., 2002) and the behaviors from intergroup affect and stereotypes (BIAS) Map (Cuddy et al., 2007) how professional men and women in high and low status positions who work in male and female-dominated occupations are perceived, as well as the affective responses and the behavioral tendencies that they arouse (See **Table 1** for theoretical background).

**Table 1:** Summary of the theoretical background and the proposed hypotheses.

Variables	Theoretical background	As a function of
**Socio-structural- variables**: Status Competition	Stereotype Content Model (SCM; Fiske et al., 2002) (Hypotheses H_1_, H_2_, H_3_, H_4_)	Professional Status Type of company Gender
**Stereotypes**: Competence and Warmth	Social Role Theory (SRT; Eagly, 1987) and Stereotype Content Model (SCM; Fiske et al., 2002) (Hypotheses H_5_, H_6_)	
**Emotions**: Admiration and Envy	Stereotype Content Model (SCM, Fiske et al., 2002) (Hypotheses H_7_, H_8_)	
**Emotions as mediating variables** between Stereotypes and Behaviors	The Behaviors from Intergroup Affect and Stereotypes (BIAS) Map (Cuddy et al., 2007) (Hypotheses H_9_, H_10_)	

### The Social Role Theory

Since the 1960s, vast research has been carried out about gender stereotypes in the workplace. However, studies which provide a fine-grained level of analysis linked to specific gender roles are within the framework of social role theory (SRT, Eagly, 1987). This perspective holds that gender stereotypes arise from three features of the social structure: (a) the gendered division of labor (employees, masculine traits and men vs. homemakers, feminine traits and women); (b) the gender hierarchy (high status roles, masculine traits and men vs. low status roles, feminine traits and women); and (c) the sex-typed distribution in paid work. In this regard, masculine traits are linked to masculine occupations (e.g., leadership positions) whereas feminine characteristics are typical of occupations symbolizing the professionalization of domestic work (e.g., secretary). SRT also argues that perceivers infer the traits of the role of occupants by observing role-constrained behavior, so when women and men perform the same role, they are perceived equivalently without any kind of gender-stereotypic judgments.

Studies conducted during this time corroborate central claims of SRT (Eagly and Steffen, 1984, 1986; Conway et al., 1996). Nevertheless, research also shows some unexpected results, probably due to the particular selection of occupations (Conway et al., 1996). Thus, a professional woman is considered as more masculine than her male counterpart regardless of the occupational status. On the other hand, women in lower status posts are perceived as more feminine than their male colleagues, whereas men and women in higher status positions do not differ in the perceived femininity (Eagly and Steffen, 1984). In the same vein men and women employees are perceived as being more masculine and less feminine in high status than in low status positions (Conway et al., 1996).

Recent studies also provide support for social role theory’s predictions (e.g., Bosak et al., 2012; Koenig and Eagly, 2014). In this vein, male and female employees are perceived as more agentic and less communal than persons without role information. In addition, male and female employees occupying a female-dominated role, or a male-dominated role are judged equally agentic and communal (Bosak et al., 2012). In high status positions, results are somewhat different. A male candidate to a leadership position is perceived as more masculine and less feminine in a masculine industry than in a feminine one (Heilman et al., 2004; García-Retamero and Lopez-Zafra, 2006). In contrast, the female candidate is perceived as equally masculine regardless of the type of company, but she scores higher in femininity when she works in a feminine industry (García-Retamero and Lopez-Zafra, 2006). The same applies to professional men behaving in a counter-stereotypical manner (e.g., modest) (Moss-Racusin et al., 2010) or working in an incongruent environment (Heilman and Wallen, 2010). For example, successful male leaders in a feminized industry are considered less competent, more ineffective and less worthy of respect and admiration than their feminine counterpart or than male leaders with the same success in a masculine industry (Heilman et al., 2004; Heilman and Wallen, 2010).

In sum, these studies reveal that in the workplace there prevails a system of segregation and gender stereotyping that enables us to link certain occupations descriptively and prescriptively to men or to women (Cabrera et al., 2009). Paradoxically, the covariation of sex and role is not completely wiped out. Women leaders are perceived as more masculine than men independent of the type of company, or more feminine in a feminine industry (García-Retamero and Lopez-Zafra, 2006). Further, successful professional men are perceived as less masculine if they work in an incongruent environment (feminine) (Heilman and Wallen, 2010).

### The Stereotype Content Model

The broad majority of research on gender discrimination in employment has focused almost exclusively on the cognitive aspects of prejudice (Schein, 1973; Eagly and Steffen, 1984). These studies have left out that (a) cognitive, affective and behavioral components of prejudice might act jointly in specific social situations; and (b) emotions might be more strongly and directly related to behavior than cognitions (Fiske et al., 2002).

On the basis of previous principles, the Stereotype Content Model (SCM, Fiske et al., 2002) and the subsequent development of the BIAS map (Cuddy et al., 2007) explain the intergrupal discrimination (e.g., ethnic, linguistic) on a general level rather than focusing exclusively on gender. The SCM holds that the basic dimensions of stereotypes are competence and warmth. Both arise out of the need to predict others’ intentions (positive vs. negative) as well as their capability to enact them. The competence dimension (e.g., intelligence) is related to the perceived ability of certain groups to accomplish their goals successfully. The warmth dimension refers to the establishment of relationships with others.

Unlike SRT, the perceived warmth and competence follow from social structural relations between groups, namely status and competition. According to this, high status groups are perceived as competent and low status groups as less competent. Non-competitive groups are viewed as warm and competitive groups as cold. Several studies have confirmed the foregoing relations (e.g., Fiske et al., 1999, 2002; Cuddy et al., 2007). The relative status of social groups is positively related to their perceived competence and it does not seem to affect judgments of warmth (Russell and Fiske, 2008). On the other hand, the perceived competitiveness is negatively related to the warmth dimension.

This model also predicts that these structural variables give rise to four combinations of stereotypes (high/low warmth by high/low competence) which, in turn, elicit unique patterns of emotions and discriminatory behavioral intentions. Specifically, groups stereotyped as competent and warm (e.g., middle class) elicit admiration and behaviors relating to helping and cooperation (active and passive facilitation). Groups characterized as incompetent and cold (e.g., opportunistic) elicit contempt and behaviors ranging from avoidance to exclusion of others (passive and active harm). On the other hand, groups stereotyped as incompetent and warm (e.g., housewives) elicit pity and behaviors ranging from helping to being dismissive (active facilitation and passive harm). Lastly, groups characterized as competent and cold (e.g., professional women) elicit envy and behaviors ranging from cooperation to action against others (passive facilitation and active harm). Furthermore, it has been shown that: (a) admiration mediates between warmth and active facilitation as well as between competence and passive facilitation; (b) contempt mediates between warmth and active harm; (c) pity mediates between competence stereotypes and active facilitation; and (d) envy does not serve as a mediator.

Central claims of the SCM have been corroborated in different countries (Cuddy et al., 2009), with diverse social groups (Cuddy et al., 2009) and feminine subtypes (e.g., Cuadrado and López-Turrillo, 2014), as well as the mediating role of intergroup emotions (Ashbrock and Cuddy, 2013, unpublished). Specifically, research conducted on different gender subgroups reveals that traditional women (housewives, women in general, and clerks) elicit pity and are viewed as low status and non-competitive as well as warm but not competent. However, the nontraditional ones (e.g., professional women) elicit envy are perceived as high status and competitive as well as competent but cold (e.g., Fiske et al., 2002; Cuadrado and López-Turrillo, 2014).

Generally, empirical work regarding the application of the SCM in the workplace shows that competence takes priority over the warmth dimension (Cuddy et al., 2011). However, factors such as parental status or type of industry moderate judgements about targets. In this vein, professional women gain in warmth with motherhood but lose in competence. Therefore, professional women are less likely to be promoted or invited to participate in continuing training than working fathers and childless workers (Cuddy et al., 2004). Research has also shown that women described with feminine job titles appear less competent and less warm and elicit more discriminatory intentions than those described with masculine job titles (Budziszewska et al., 2014).

Summarizing, in comparative contexts gender subgroups are characterized in terms of compensation (contrast effect) (Kervyn et al., 2008) in such a way that some subtypes of women are perceived as warm and incompetent and others as competent and cold. In the organizational context, however, professionals are rated in terms of the halo effect, such as positive information on competence leading to positive judgements on the warmth dimension. In addition, it should be noted that SRT and SCM are not mutually exclusive. The former analyzes the perceivers’ more elementary observations of group members in their typical roles whereas the latter emphasizes broad, molar social structural correlates of stereotypes (Koenig and Eagly, 2014). Furthermore, it should be taken into consideration that the traditional gendered division of labor is implicitly based on interdependence (women depending on men as providers and men on women as homemakers) and status (high status roles and men vs. low status roles and women), whereby these stereotypes can also be seen as complementary of the SCM (Fiske et al., 2002).

### The Current Research

The main focus of this study is to examine from the SCM (Fiske et al., 2002) and the BIAS map (Cuddy et al., 2007) whether cognitive, affective and behavioral components of prejudice act jointly to explain gender segregation in the labor market. With this, we intend to find out whether emotions elicited are the key proximal influence by which stereotypes of men and women in traditional and non-traditional occupations are translated into actions (Cuddy et al., 2007). With this aim in mind, our sample will be made up exclusively of people with work experience. The supposition is that they have clearer norms about the behaviors needed to successfully perform the professional role (Eagly et al., 2000) than people without work experience (e.g., students). To avoid problems with the operationalization of occupations (Conway et al., 1996), we used the roles of leader and secretary as comparison targets because both occupations (a) are the most highly masculinized and feminized in all countries (European Commission, 2009), and (b) have acquired certain stereotypical traits (e.g., task vs. supporting-others oriented) that single them out as “men’s work” and “women’s work,” respectively (Schein, 1973; Truss et al., 1995). Moreover, given that the maleness or femaleness of a working context depends on the perception that a greater proportion of men or women work in that environment (Pazy and Oron, 2001), we select two working areas with a high percentage of men (high technology) and women (health) (European Commission, 2009). With all this we aim to reduce the stereotypical judgements about the professional roles evaluated (to remove the covariation of sex and role).

On the basis of the foregoing considerations, we analyzed workers’ perception of men and women professionals with different occupational status (leader vs. secretary) who work in masculine (high technology) and feminine (health) domains, as well as the behavioral tendencies aroused. According to the SCM and the BIAS map, we suppose that leaders will be perceived as having higher status and as being more competitive than secretaries. Furthermore, based on the premises of SRT (Eagly, 1987), we assume that their ratings on status and competition will only vary depending on the type of company, but not on gender. Accordingly, we expect that:

*Hypothesis 1*. Leaders (men and women) will be perceived as having higher status than secretaries (men and women).
*Hypothesis 2*. Leaders and secretaries (men and women) will be perceived as having more status in the high-tech than in the health company.
*Hypothesis 3*. Leaders (men and women) will be perceived as being more competitive than secretaries (men and women).
*Hypothesis 4*. Leaders and secretaries (men and women) will be perceived as being more competitive in the high-tech than in the health company.

Given that the SCM and the BIAS map state that warmth and competence follow from social structural relations between groups, namely status and competition, we expect that male and female leaders (high status/high competition) will be perceived as more competent and less warm than male and female secretaries (low status and less competitive). Likewise, we suppose that ratings on perceived competence and warmth (male and female leaders and secretaries) will vary as a function of the type of company but not of gender (e.g., Eagly, 1987). In accordance with this, we expect that:

*Hypothesis 5*. Leaders (men and women) will be perceived as more competent and less warm than secretaries (men and women).
*Hypothesis 6*. Leaders and secretaries (men and women) will be perceived as more competent and less warm in the high-tech than in the health company.

From the SCM and the BIAS map, it logically follows that neither leaders nor secretaries are likely to elicit contempt (incompetent and cold stereotypes) or pity (incompetent and warm stereotypes), given that in the organizational context, competence stereotypes take priority over warmth stereotypes (e.g., Cuddy et al., 2011). In the same vein, we assume that professional men and women will elicit more admiration and envy in high than in low status positions, as well as in the high-tech compared to the health company. In accordance with this, we expect that:

*Hypothesis 7*. Leaders (men and women) will elicit more admiration and envy than secretaries (men and women).
*Hypothesis 8*. Leaders and secretaries (men and women) will elicit more admiration and envy in the high-tech than in the health company.

Finally, we intend to prove whether in the organizational context emotions more strongly and directly predict behaviors than stereotypes. From the BIAS map (Cuddy et al., 2007), we suppose that admiration and/or envy will mediate the direct effect of the perceived competence of men and women leaders on passive facilitation, especially when they work in the high-tech company. For active facilitation, however, admiration will mediate the effect of the perceived warmth of secretaries, in particular when they work in the health company. In accordance with the aforementioned, we expect that:

*Hypothesis 9*. Admiration and envy will mediate the direct effect of the perceived competence of men and women leaders on passive facilitation, especially in the high-tech company.
*Hypothesis 10*. Admiration will mediate the direct effect of the perceived warmth of men and women secretaries on active facilitation, especially in the health company.

## Materials and Methods

### Participants

The sample consisted of 1089 Spanish participants (513 men and 576 women) with work experience, ranging from 18 to 76 years old (*M* = 38.75; *SD* = 10.74). Fifty per cent of them (50.32%) had higher education (*n* = 548) and the remainder were distributed into primary (*n* = 92), secondary (*n* = 272) and vocational education (*n* = 142). Participants differed in employment status and had different occupations [categorized according to International International Labour Organization [ILO], 2007]: managers (*n* = 40), professionals (*n* = 310), technicians (*n* = 168), clerical support (*n* = 198), service and sales (*n* = 136), skilled agricultural (*n* = 3), craft workers (*n* = 70), machine operators (*n* = 8), elementary occupations (*n* = 48), and armed force occupations (*n* = 11). Finally, 35% of workers (*n* = 390) had held leadership positions over the course of their professional life.

### Procedure

Questionnaires were collected over a period of 8 months. Students on final courses of Psychology and Social Work cooperated voluntarily in exchange for extra course credits. Persons with employment experience were contacted by e-mail (and reminder mailing). The recruitment procedure was online. Thus, participants completed the registration page and the consent form first and filled out the questionnaire second. Anonymity and confidentiality were guaranteed. In order to avoid bias and repeated participation, participants who did not complete their personal data or the consent form were removed from the data base (*n* = 206).

### Instrument

To carry out our goals, a questionnaire with eight versions was designed. The first four conditions focused on professionals in high status positions who worked in male or female-dominated occupations: A man (Version 1, *n*_men_ = 67 vs. *n*_women_ = 75) and a woman (Version 2, *n*_men_ = 64 vs. *n*_women_ = 67) chief executive officer in a high-tech company, and a man (Version 3, *n*_men_ = 57 vs. *n*_women_ = 68) and a woman (Version 4, *n*_men_ = 57 vs. *n*_women_ = 68) chief executive officer in a company dedicated to health services. The last four versions of the questionnaire concerned professionals in low status posts: A man (Version 5, *n*_men_ = 69 vs. *n*_women_ = 78) and a woman (Version 6, *n*_men_ = 71 vs. *n*_women_ = 76) as administrative secretary in a high-tech company, and a man (Version 7, *n*_men_ = 51 vs. *n*_women_ = 66) and a woman (Version 8, *n*_men_ = 57 vs. *n*_women_ = 66) as administrative secretary in a company dedicated to health services.

Participants were randomly assigned to one of the eight versions. They were told that they were taking part in a study on the working world. After reading the instructions, they were requested to rate the particular target. The questionnaire used the items proposed in the SCM (Fiske et al., 2002, Study 2) or the BIAS map (Cuddy et al., 2007) adapted to the Spanish. Scales were presented in the following order.

#### Socio-Structural Dimensions

Scales from Fiske et al. (2002) were adapted to the working world. The perceived status was measured by two items: (a) “In your opinion, how prestigious are the jobs typically achieved by a (target)?” and (b) “In your opinion, how economically successful is a (target)?” (α = 0.77). Scales ranged from 1 (*not at all*) to 5 (*a lot*). Likewise, the perceived competitiveness was also assessed by two items: (a) “If a (target) gets special breaks, such as preference in hiring decisions, this is likely to make things more difficult for people like me,” and (b) “Resources that go to a (target) are likely to take away from the resources of people like me” (α = 0.66). Scales ranged from 1 (*totally disagree*) to 5 (*totally agree*).

#### Stereotypes

To measure the stereotype content, scales adapted from Fiske et al. (2002) by Cuadrado and López-Turrillo (2014) were used. Participants rated the attributes of targets in response to the question, “In your opinion, how typical are the following attributes of a (target)?” Scales ranged from 1 (*not at all*) to 5 (*a lot*). The competence subscale included six adjectives (competent, confident, capable, efficient, intelligent, and skillful; α = 0.88). The warmth subscale also contained six items (friendly, well-intentioned, trustworthy, warm, good-natured, and sincere; α = 0.89).

#### Emotions

Likewise, to measure the emotions, scales adapted from Fiske et al. (2002) by Cuadrado and López-Turrillo (2014) were used. Participants expressed the degree to which they felt a set of 17 emotions in relation to the target, using scales ranging from 1 (*never*) to 5 (*always*). As proposed by SCM (Fiske et al., 2002), emotional hypotheses build on social comparison-based (Smith, 2000) and attributional (Weiner, 2005) models of emotions. Following Fiske et al. (2002) as well as the correlational (Cuddy et al., 2007) and empirical evidence (e.g., Caprariello et al., 2009; Cuadrado and López-Turrillo, 2014), emotions was grouped into four variables: admiration (admiring, fond, inspired, proud, and respectful; α = 0.82), envy (envious and jealous; α = 0.85), contempt (angry, ashamed, contemptuous, disgusted, frustrated, hateful, resentful, and uneasy; α = 0.89), and pity (pitying and sympathetic; α = 0.26). The pity variable was dropped from the analysis because of its low reliability. One main cause seemed to be that this variable shares little in common with other variables in the domain of interest (Fabrigar et al., 1999). That is, a professional context likely primes high competence (vs. low warmth), which, in turn, elicit admiration and envy (Cuddy et al., 2007).

#### Behaviors

The behavioral tendencies were measured by four items adapted to the working world from Cuddy et al. (2007) using scales ranged from 1 (*not at all*) to 5 (*a lot*): (a) “I would cooperate with (target)” (passive facilitation); (b) “I would protect and help the (target)” (active facilitation); (c) “I would fail to recognize his/her worthiness” (passive harm); and (d) “I would undermine his/her work” (active harm).

#### Socio-Demographic and Employment Data

Participants indicated their sex, age, educational level, labor status, job type, and experience in management.

### Data Analyses

First, we carried out two 2 × 2 × 2 analyses of variance to analyze the effect of occupational status (high vs. low), type of company (high-tech vs. health) and workers’ sex (men vs. women) on the social structural variables (status and competition). Thereafter, two ANOVAs with repeated measures were performed to examine the effect of occupational status, type of company and workers’ gender on the stereotyped dimensions (competence and warmth) and on emotions (admiration, envy, and contempt), with the first three factors as between-participant variables and the latter two as within-participant variables. In all cases, variance homogeneity assumption was verified by Levene’s test (*p* > 0.05). Tukey and Bonferroni tests were applied to analyze multiple comparisons. Finally, a series of mediational analyses using the PROCESS macro for SPSS (Hayes, 2013) was carried out in order to examine the link between stereotypes, emotions, and behavioral tendencies.

## Results

### Socio-Structural Variables: Status and Competition

The ANOVA performed on the status variable yielded a main effect of occupational status, *F*(1,1086) = 281.47, *p* < 0.001, η_p_^2^ = 0.21, as workers ascribed more status to professionals in leadership positions than in administrative posts. The analysis also revealed a main effect of workers’ sex, *F*(1,1086) = 8.77, *p* = 0.003, η_p_^2^ = 0.008, such that participants reported that professional women possess more status than professional men. These results were qualified by the two–way interaction between occupational status and workers’ sex, *F*(1,1086) = 20.78, *p* < 0.001, η_p_^2^ = 0.019. *Post hoc* tests revealed that participants ascribed women secretaries more status than men secretaries (*p* < 0.001), whereas their status ratings did not differ when they evaluated men and women leaders (*p* = 0.25) (see **Table 2**). These results partially confirmed Hypothesis 1, but not Hypothesis 2.

**Table 2:** Means and standard deviations of socio-structural variables status and competition by occupational status, type of company, and worker’s sex.

	High status posts Leaders			Low status posts Secretaries			Total		
	Man *M* (*SD*)	Woman *M (SD)*	Total *M (SD)*	Man *M (SD)*	Woman *M (SD)*	Total *M (SD)*	Man *M (SD)*	Woman *M (SD)*	Total *M (SD)*
	**Status (a)**								
High-tech	3.81 (0.72)	3.67 (0.84)	3.74 (0.78)	2.62 (0.97)	3 (0.96)	2.81 (0.98)	3.20 (1.04)	3.31 (0.96)	3.25 (1)
Health	3.69 (0.85)	3.65 (0.83)	3.66 (0.84)	2.44 (0.96)	2.91 (1.21)	2.68 (1.17)	3.08 (1.16)	3.32 (1.08)	3.21 (1.12)
Total	3.75 (0.79)	3.66 (0.83)	3.70 (0.81)	2.54 (1.03)	2.96 (1.08)	2.75 (1.08)	3.15 (1.09)	3.32 (1.02)	3.24 (1.06)
	**Competition (b)**								
High-tech	2.44 (1.09)	1.98 (1.03)	2.23 (1.08)	1.80 (1.03)	1.75 (1.11)	1.77 (1.07)	2.12 (1.10)	1.86 (1.08)	1.99 (1.10)
Health	2.63 (1.29)	2.37 (1.31)	2.48 (1.30)	2.03 (1.29)	2.13 (1.27)	2.08 (1.28)	2.34 (1.32)	2.26 (1.30)	2.30 (1.31)
Total	2.53 (1.18)	2.20 (1.20)	2.36 (1.21)	1.90 (1.16)	1.92 (1.20)	1.91 (1.18)	2.22 (1.21)	2.06 (1.21)	2.14 (1.21)

*Scores range from (a) 1 (not at all) to 5 (a lot), (b) 1 (totally disagree) to 5 (totally agree).*

The ANOVA of the competition scale revealed a main effect of occupational status, *F*(1,1086) = 35.99, *p* < 0.001, η_p_^2^ = 0.032. Competition was rated as being more characteristic for leadership positions than for a clerical post. Moreover, there was a main effect of type of company, *F*(1,1086) = 16.89, *p* < 0.001, η_p_^2^ = 0.015, reflecting that competition was perceived as more typical of the health than of the high-tech company. Additionally, the analysis yielded a single effect of workers’ sex, *F*(1,1086) = 5.40, *p* = 0.020, η_p_^2^ = 0.004, such that participants considered men workers more competitive than women workers. In the light of the preceding main effect, we also found an interaction between occupational status and workers’ sex, *F*(1,1086) = 6.97, *p* = 0.008, η_p_^2^ = 0.006. Bonferroni tests revealed that competition was evaluated as more characteristic of the men leaders than of the women leaders, *p* < 0.001. For men and women secretaries, no differences emerged (*p* = 0.83). Likewise, these data partially confirmed Hypothesis 3, but not Hypothesis 4.

### Stereotypes: Competence and Warmth

The repeated measures ANOVA yielded a significant main effect of the stereotypes, *F*(1,1081) = 348.09, *p* < 0.001, η_p_^2^ = 0.24; of the occupational status, *F*(1,1081) = 11.53, *p* < 0.001, η_p_^2^ = 0.011; of the type of company *F*(1,1081) = 39.76, *p* < 0.001, η_p_^2^ = 0.035; and of workers’ sex, *F*(1,1081) = 41.91, *p* < 0.001, η_p_^2^ = 0.037. As can be seen in **Table 3**, *post hoc* analyses revealed that participants rated all targets as more competent than warm. Similarly, they evaluated leaders more positively than secretaries, professionals in the high-tech company more positively than those in the health company, and women workers more positively than men workers.

**Table 3:** Means and standard deviations of competence and warmth by occupational status, type of company, and worker’s sex.

	High status posts Leaders			Low status posts Secretaries			Total		
	Man *M* (*SD*)	Woman *M (SD)*	Total *M (SD)*	Man *M (SD)*	Woman *M (SD)*	Total *M (SD)*	Man *M (SD)*	Woman *M (SD)*	Total *M (SD)*
	**Competence**								
High-tech	3.71 (0.59)	3.89 (0.63)	3.80 (0.62)	3.30 (0.72)	3.66 (0.60)	3.48 (0.69)	3.50 (0.69)	3.77 (0.63)	3.63 (0.67)
Health	3.40 (0.79)	3.61 (0.67)	3.52 (0.73)	3.00 (0.71)	3.16 (0.79)	3.08 (0.76)	3.21 (0.78)	3.41 (0.76)	3.32 (0.77)
Total	3.56 (0.70)	3.74 (0.67)	3.65 (0.69)	3.17 (0.73)	3.43 (0.74)	3.30 (0.75)	3.37 (0.75)	3.59 (0.72)	3.48 (0.74)
	**Warmth**								
High-tech	2.71 (0.93)	3.20 (0.88)	2.95 (0.94)	3.11 (0.83)	3.43 (0.77)	3.27 (0.81)	2.91 (0.90)	3.32 (0.83)	3.11 (0.89)
Health	2.84 (0.96)	3.15 (0.87)	3.01 (0.92)	2.80 (0.93)	2.93 (0.88)	2.87 (0.91)	2.82 (0.94)	3.06 (0.88)	2.95 (0.92)
Total	2.77 (0.95)	3.17 (0.88)	2.98 (0.93)	2.97 (0.89)	3.20 (0.85)	3.09 (0.88)	2.87 (0.92)	3.19 (0.86)	3.03 (0.91)

*Scores range from 1 (not at all) to 5 (a lot).*

Additionally, the analysis also yielded three two–way interactions among (a) occupational status and type of company, *F*(1,1081) = 10.40, *p* < 0.001, η_p_^2^ = 0.04; (b) stereotypes and occupational status, *F*(1,1081) = 95.504, *p* < 0.001, η_p_^2^ = 0.08; and (c) stereotypes and type of company, *F*(1,1081) = 11.96, *p* < 0.001, η_p_^2^ = 0.04. With respect to the Occupational Status × Type of Company interaction, results showed that leaders and secretaries in the high-tech company were equally valued (*p* = 0.90), whereas in the health company, leaders were more positively evaluated than secretaries (*p* < 0.001).

With regard to the Stereotypes × Occupational Status interaction, *post hoc* analysis indicated that competence was perceived as more characteristic of leaders than of secretaries (*p* < 0.001), whereas no differences emerged in the warmth dimension (*p* = 0.09). These data partially confirmed Hypothesis 5. In relation to the Stereotypes × Type of Company interaction, Bonferroni tests revealed that participants attributed more competence and warmth to professionals in the high-tech company than to those in the health company (*p* < 0.001).

Finally, we found a three–way interaction between occupational status, stereotypes and workers’ sex, *F*(1,1081) = 6.19, *p* = 0.013, η_p_^2^ = 0.006, and another one between occupational status, stereotypes and type of company, *F*(1,1081) = 13.14, *p* < 0.001, η_p_^2^ = 0.012. Bonferroni tests relative to the Occupational Status × Stereotypes × Worker’s Sex interaction reflected that men leaders were evaluated as more competent (*p* < 0.001) and less warm (*p* = 0.02) than men secretaries, whereas women leaders were perceived as more competent than women secretaries (*p* < 0.001), but warm to the same extent (*p* = 0.97). Regarding the Occupational Status × Stereotypes × Type of Company interaction, results partially confirmed Hypothesis 6. In this vein, Bonferroni tests showed that in the high-tech company, leaders were evaluated as more competent and less warm than secretaries (all *p_s_* < 0.001), whereas in the health company, leaders were perceived as more competent than secretaries (*p* < 0.001) and equally warm (*p* = 0.09). Furthermore, leaders were rated as more competent in the high-tech company than in the health company (*p* < 0.001), but warm to the same extent (*p* = 0.60). In contrast, secretaries were evaluated as more competent and warmer in the high-tech than in the health company (all *p_s_* < 0.001).

### Emotions: Admiration, Envy, and Contempt

The repeated measures ANOVA produced a significant main effect of emotions, *F*(2,1081) = 867.88, *p* < 0.001, η_p_^2^ = 0.45; occupational status, *F*(1,1081) = 13.09, *p* < 0.001, η_p_^2^ = 0.012; workers’ sex, *F*(1,1081) = 14.73, *p* < 0.001, η_p_^2^ = 0.013; and type of company, *F*(1,1081) = 4.42, *p* = 0.041, η_p_^2^ = 0.004. As shown in **Table 4**, Bonferroni tests indicated that all targets elicited more admiration than envy and contempt (*p* < 0.001). No differences emerged between envy and contempt (*p* = 0.06). Likewise, leaders as well as professional women and workers in the high-tech company compelled more affective responses than secretaries, professional men and those in the health company, respectively.

**Table 4:** Means and standard deviations of the emotions admiration, envy, and contempt by occupational status, type of company, and worker’s sex.

	High status posts Leaders			Low status posts Secretaries			Total		
	Man *M* (*SD*)	Woman *M (SD)*	Total *M (SD)*	Man *M (SD)*	Woman *M (SD)*	Total *M (SD)*	Man *M (SD)*	Woman *M (SD)*	Total *M (SD)*
	**Admiration**								
High-tech	2.33 (0.81)	2.76 (0.73)	2.54 (0.80)	2.16 (0.73)	2.45 (0.87)	2.30 (0.81)	2.24 (0.77)	2.60 (0.83)	2.42 (0.82)
Health	2.15 (0.83)	2.50 (0.86)	2.35 (0.86)	2.07 (0.84)	2.23 (0.80)	2.15 (0.82)	2.11 (0.83)	2.38 (0.84)	2.26 (0.85)
Total	2.24 (0.82)	2.62 (0.81)	2.44 (0.84)	2.12 (0.78)	2.35 (0.85)	2.24 (0.82)	2.18 (0.80)	2.49 (0.84)	2.34 (0.83)
	**Envy**								
High-tech	1.57 (0.83)	1.60 (0.94)	1.58 (0.88)	1.32 (0.67)	1.49 (0.85)	1.40 (0.77)	1.44 (0.76)	1.54 (0.89)	1.49 (0.83)
Health	1.42 (0.64)	1.43 (0.75)	1.42 (0.70)	1.32 (0.82)	1.42 (0.67)	1.37 (0.74)	1.37 (0.73)	1.43 (0.71)	1.40 (0.72)
Total	1.50 (0.75)	1.51 (0.84)	1.50 (0.80)	1.32 (0.74)	1.46 (0.77)	1.39 (0.76)	1.41 (0.75)	1.48 (0.81)	1.45 (0.78)
	**Contempt**								
High-tech	1.45 (0.65)	1.40 (0.70)	1.43 (0.67)	1.25 (0.40)	1.37 (0.64)	1.31 (0.54)	1.35 (0.55)	1.38 (0.67)	1.37 (0.61)
Health	1.41 (0.68)	1.45 (0.72)	1.43 (0.70)	1.45 (0.71)	1.41 (0.54)	1.43 (0.63)	1.43 (0.70)	1.43 (0.64)	1.43 (0.67)
Total	1.43 (0.67)	1.42 (0.71)	1.43 (0.69)	1.34 (0.57)	1.39 (0.59)	1.37 (0.58)	1.39 (0.62)	1.41 (0.66)	1.40 (0.64)

*Scores range from 1 (never) to 5 (always).*

The analyses also yielded three two–way interactions among (a) occupational status and emotions, *F*(2,1081) = 4.85, *p* = 0.008, η_p_^2^ = 0.004; (b) workers’ sex and emotions, *F*(2,1081) = 18.79, *p* < 0.001, η_p_^2^ = 0.017; and (c) type of company and emotions, *F*(2,1081) = 12.43, *p* < 0.001, η_p_^2^ = 0.011. Regarding the Occupational Status × Emotions interaction, Bonferroni tests showed that leaders elicited more envy than contempt (*p* = 0.02), whereas secretaries elicited both emotions to the same extent (*p* = 1). Besides these findings, results partially confirmed our Hypothesis 7: leaders elicited more admiration (*p* < 0.001) and envy (*p* = 0.02) than secretaries. No differences emerged concerning contempt (*p* = 0.17). In relation to the Workers’ Sex × Emotions interaction, *post hoc* analysis revealed that professional women elicited more admiration than professional men (*p* < 0.001). With regard to the Type of Company × Emotions interaction, results of Bonferroni tests confirmed the Hypothesis 8: the high-tech company compelled more envy than contempt (*p* < 0.001), whereas the health company elicited these emotions in equal measure (*p_s_* > 0.05). It is also worth noting that the high-tech company elicited more admiration (*p* < 0.001) and envy (*p* = 0.04) than the health company. No differences emerged concerning contempt (*p* = 0.11).

Lastly, the analysis showed a three–way interaction between emotions, workers’ sex and occupational status, *F*(2,1081) = 4.40, *p* = 0.012, η_p_^2^ = 0.004. Bonferroni tests revealed that women leaders compelled more admiration than women secretaries (*p* < 0.001), and envy and contempt to the same extent (*p* > 0.05). In contrast, men leaders elicited more envy than men secretaries (*p* = 0.011), whereas no differences emerged concerning admiration and contempt (*p* > 0.05). Furthermore, women leaders elicited more admiration than men leaders (*p* < 0.001), and envy and contempt to the same extent (*p* > 0.05), whereas women secretaries compelled more admiration (*p* = 0.002) and more envy (*p* = 0.044) than the men secretaries, and contempt to the same extent (*p* > 0.05).

### Differences in the Variables Studied According to the Sex of the Participant

The analyses conducted showed that there were no significant differences according to participants’ sex in any of the variables measured (*p* > 0.05).

### Mediation Analyses

To test whether emotions prevailed over stereotypes in predicting behavioral tendencies, four mediation analyses with bootstrapping procedures were performed using the PROCESS macro for SPSS (Hayes, 2013). In particular, we used bootstrapping methodology (Preacher and Hayes, 2004) with 10,000 resamples to estimate 95% confidence intervals for the indirect effects of stereotypes (i.e., competence or warmth as independent variables) on behavioral tendencies (i.e., active facilitation, passive facilitation, active harm, and passive harm as criterion variables) by emotions (i.e., admiration, envy, and contempt as mediators). In all analyses, the effects of the non-predictor trait (competence or warmth), the workers’ sex, the occupational status and the type of company were controlled by adding them into the model as covariates. As prescribed in this procedure, an indirect effect is significant where zero is not contained in the 95% confidence interval.

**FIGURE 1:**
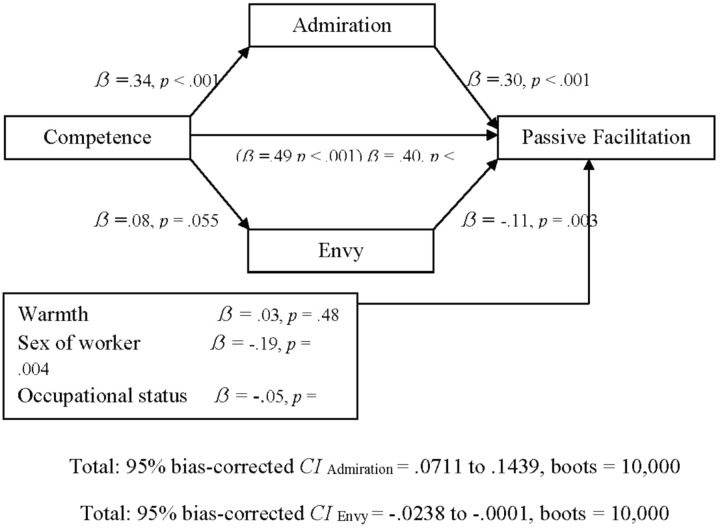
Standardized β coefficients (in parentheses), and standardized β coefficients reduced when admiration and envy are introduced as mediating variables between competence and passive facilitation and the covariates warmth, worker’s sex, occupational status and type of company. The significant covariates are highlighted in bold. CI, Confidence Interval.

**FIGURE 2:**
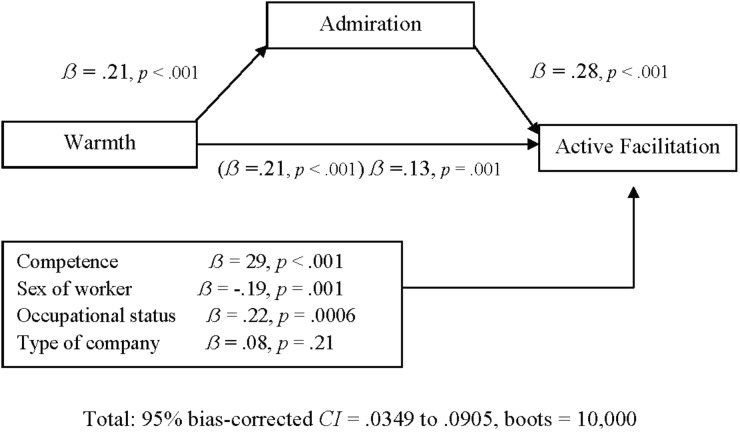
Standardized β coefficients (in parentheses), and standardized β coefficients reduced when admiration is introduced as a mediating variable between warmth and active facilitation, and the covariates warmth, sex of worker’s sex, occupational status and type of company. The significant covariates are highlighted in bold. CI, Confidence Interval.

As presented in **Figures 1**, **2**, results partially confirmed Hypotheses 9 and 10. Admiration and envy partially mediated the direct effect of competence on passive facilitation. The effect of worker’s sex (men) remained significant when admiration was included in the equation. On the other hand, admiration also partially mediated the effect of warmth on active facilitation. Moreover, the effect of the competence, worker’s sex (men) and occupational status (low) remained significant when admiration was added into the model. Finally, envy and contempt were not significant mediators of the effect of stereotypes on the behaviors of passive and active harm, because zero was contained within their respective confidence intervals.

## Discussion

The main focus of this study was to examine whether cognitive, affective and behavioral components of prejudice could act jointly to explain the discrimination of women and men who work in traditional and non-traditional gender-linked roles. Overall, relative status and perceived competition are particularly relevant in determining stereotypes of both leaders and secretaries and consequently emotions evoked and behavioral tendencies elicited (Cuddy et al., 2007), especially for professional men.

Regarding our first four hypotheses, data indicate that leaders as well as professional women are perceived as possessing more status than secretaries and professional men. Moreover, men and women leaders do not differ in their perceived status, whereas women secretaries are viewed as possessing more status than their male counterparts. Results related to perceived competition show a slightly different pattern. Competition is ascribed to leaders as well as men workers and professionals in the health company to a greater extent than to secretaries, women workers, and those professionals in the high-tech company. Furthermore, men leaders are considered more competitive than women leaders, whereas men and women secretaries do not differ in their perceived competitiveness. All these results taken together support the basic tenets of SCM (Fiske et al., 2002): In occupational hierarchies, leaders are associated with more status and competition than secretaries. Nevertheless, contrary to our expectations, judgments on status and competition seem to depend on the sex-stereotypicality of the target. In this vein, our results suggest that workers perceive that female leaders have the same ability to control and regulate economic and human resources (status) but less intention of optimizing them than their male counterparts (competition). At an interpersonal level, however, it implies that workers consider female leaders to pose less of a threat to their professional interests such as promotions, professional training or increases in pay. As a result, with the same status, men leaders retain their advantage over women leaders to manage and to compete more successfully, regardless of type of company. In low status positions, however, women maintain their “advantage” because men violate gender-based expectations that require them to possess higher status than women (Moss-Racusin et al., 2010).

With respect to stereotypes, the most interesting results concern warmth and competence dimensions and their linkage with the variables analyzed. In this sense, all professionals are construed primarily in terms of higher competence and less warmth. Nevertheless, our prediction is partially fulfilled (H5). Leaders are perceived as considerably more competent than secretaries, but equally warm. As other studies suggest (Eagly and Steffen, 1984; Cuddy et al., 2004, 2011; Duehr and Bono, 2006), competence takes primacy over warmth judgments within the organizational context, inasmuch as normative behaviors to perform the professional role successfully are stereotypically masculine (e.g., Eagly et al., 2000), especially in high status positions (e.g., Glick et al., 2005). On the other hand, the fact that leaders and secretaries do not differ in the perceived warmth may be related to the decrease in the construed masculinity of managerial stereotypes toward a more androgynous view (Kark et al., 2012). Our results also reveal intra-gender differences (among professional men and among professional women). That is, professional men are evaluated in terms of mixed stereotypes (contrast effect), whereby greater competence and less warmth attributed to men leaders lead to less competence and more warmth assigned to men secretaries. On the contrary, women leaders are perceived as more competent than women secretaries and warm to the same extent. To explain these unexpected findings we also have to bear in mind that women leaders have been previously rated as being less competitive than men leaders, and, according to SCM (e.g., Fiske et al., 2002; Cuddy et al., 2011) lower rated competition may have increased the rated warmth of women leaders.

Data also show that the perception of professionals depends on occupational status and type of company, but not of gender (SRT, Eagly, 1987), although in a different way as predicted (H6). Professionals in the high-tech company are assigned more competence and warmth than those in the health company. Moreover, leaders are rated as more competent in the high-tech than in the health company, but warm to the same extent, whereas secretaries are evaluated as more competent and warmer in the high-tech than in the health company. Likewise, data also show a pattern of mixed stereotypes, but restricted to the high-tech company. In this vein, leaders in the high-tech company are viewed as more competent and less warm than secretaries, whereas leaders in the health company are perceived as more competent than secretaries and equally warm. In accordance with SRT (Eagly, 1987), male-dominated occupations are linked to typically masculine traits (competence), but also to feminine characteristics (warmth), possibly because they are perceived as a “reference” within the organizational context. One proof of this is that in the high-tech company, leaders gain perceived competence and maintain perceived warmth, while secretaries increase their perceived competence as well their perceived warmth (*halo effect*). It is also noteworthy that in contrast with previous studies (e.g., García-Retamero and Lopez-Zafra, 2006; Cabrera et al., 2009), our results do not indicate gender differences in high status positions. One possible explanation is that occupations in the aforementioned studies differ not only in their masculine and feminine demands (Conway et al., 1996), but also in their perceived status.

In regard to emotions, results partially confirm Hypothesis 7 and fully Hypothesis 8. Leaders (as well as professional women and professionals in the high-tech company) evoke significantly more admiration than secretaries, professional men and professionals in the health company. Moreover, the former also elicit more envy than contempt, whereas the latter evoke both emotions to the same extent. Nevertheless, women leaders also elicit more admiration than men leaders and envy and contempt to the same extent, whereas women secretaries evoke more admiration and more envy than the men secretaries, and contempt to the same extent. Meanwhile, the fact that professional women elicit more admiration than professional men could be seen as an acknowledgement of women’s leadership whenever it is translated into behavioral tendencies favoring professional women. Nevertheless, it does not appear that this is always the case, as will be discussed further below.

Finally, our results also show intra-gender differences. Women leaders elicit more admiration than women secretaries perhaps because they have gained a status (due to their high level of competence) inconsistent with the expectations held traditionally about women as a group (e.g., homemakers and low status jobs). But the fact that women leaders and secretaries evoke the same feelings of envy suggests that women leaders present less of a threat to men in leadership positions for being less competitive and hence warmer. In turn, men leaders and secretaries (previously evaluated in terms of mixed stereotypes) evoke the same degree of admiration due to traits related to paid productive work and especially to leadership positions (masculine) which overlap with stereotypical masculine characteristics and men (Eagly and Steffen, 1984; Eagly, 1987). Most importantly, for men leaders, admiration also co-exists with envy, which fits with mixed emotions ascribed to high-status and competitive groups (Fiske et al., 2002; Cuddy et al., 2011).

Regarding the mediating role of emotions, results pin down the aforementioned one. Admiration and envy (partially) mediate the direct effect of competence on passive facilitation (cooperate), especially for professionals in hig-status positions and men. On the other hand, admiration (partially) also mediates the effect of warmth on active facilitation (protect), above all in the case of professionals in low status positions and men, whenever they are competent. In line with the BIAS map (Cuddy et al., 2007), our findings are consistent with predictions about the effects of emotions on behaviors in ways that are consistent with perceived stereotypical traits and abilities of professionals in high (competent) and low status (warm but also competent) positions. That is to say, admiration of high competence leaders may not only increase the desire of subordinates to work closely with leaders (passive facilitation) but is also an unconscious way of recognizing their leadership (García-Ael, 2015).

Furthermore, emotions (admiration and envy) can trigger positive interpersonal effects in subordinates. Considering that admiration is a source of motivation to emulate role models (e.g., Haidt and Keltner, 2004), subordinates could be motivated to improve their own skills or to demonstrate competence in achieving goals (Galliani and Vianello, 2012) in order to gain favorable judgements from their leaders. Furthermore, admiring displayed warmth could also promote behaviors relevant to peer relationship formation (Algoe and Haidt, 2009) (e.g., cooperating with one another), while elicited envy would serve to protect workers’ self-esteem in relation to competent competitors who achieve relatively superior outcomes (Fiske et al., 2002).

Unlike our proposals, these predictions exclusively target professional men, regardless of occupational status or type of company. In this vein, our findings seem to confirm that the admired qualities in leadership are stereotypically linked to masculine traits and men (think manager – think male stereotype, Schein, 1973), which would contribute to maintain and perpetuate vertical segregation. This marked pro-male bias also exists in low status positions, paradoxically with the warmth dimension. A plausible explanation is that the perceived warmth in professional men is associated with instrumental grounds and in professional women is confused with concern (i.e., kindness) for colleagues (Cuddy et al., 2011).

Finally, we have also to note that this stereotypical view of jobs and leadership roles may be culturally mediated. Research on this topic carried out in Spain shows that leadership roles are strongly male-typed even by real workers (Cuadrado et al., 2015). As a result, Spanish professional women continue to emulate masculine behavior (Cuadrado et al., 2004), regardless of type of industry (García-Retamero and Lopez-Zafra, 2006), even though they obtain unfavorable evaluations for displaying male-stereotypical leadership styles (Cuadrado et al., 2008) and have fewer opportunities than men to develop their professional career in leadership positions (Sarrió et al., 2002). This stereotypical perception of job roles by real workers would explain why in Spain vertical and horizontal segregation is higher that the European average, and why female representation in leadership positions is still much lower than that of another European countries (European Commission, 2013), and particularly that of the United States (Catalyst Survey, 2014).

Summarizing, as proposed by SCM and the BIAS map (Fiske et al., 2002; Cuddy et al., 2007), results reveal that cognitive, affective and behavioral components of prejudice act jointly to explain discrimination against women in the workplace. In this vein, intergroup bias toward professionals varies more as a function of occupational status than sex of worker and type of company. Accordingly, gender stereotypes of men (competent) complement role stereotypes for the workforce and those for leadership positions, whereas gender stereotypes of women (warmth) not only conflict with leadership roles, but they seem to be the most suitable for low prestige jobs. Moreover, competition also seems to be critical to “exclude” women from leadership roles (less competitive and more warmth). In addition, emotions evoked are the key proximal influence by which professional stereotypes are translated into behavioral tendencies (Cuddy et al., 2007).

### Strengths, Limitations, and Future Research

This study has some limitations that need to be taken into consideration and which should be addressed in future research. The first limitation is related to the operationalization of dependent variables. In this vein, we adapted the subscales of the SCM (Fiske et al., 2002), developed to study intergroup perception and to analyse interpersonal perception. Secondly, behavioral tendencies are measured by single item variables and, in addition, are presented in a generic form that does not allow us to draw inferences about real behaviors favoring or disfavoring professional women and men. Finally, we are also aware that some of the items integrating structural variables make sense in an intergroup context, but they are less obvious at the interpersonal level. For example, perceiving competitiveness between goals of the in-group and out-group is not the same as perceiving competitiveness between goals of leaders (and, by extension, of organizations) and those of self.

Besides the fact that the results should be interpreted with caution, we must bear in mind that our sample is wholly made up of workers belonging to different organizations along with a wide range of age, education level and occupational status. Moreover, behavioral measures not only satisfy the established criteria proposed by Rossiter (2002) regarding singularity and consciousness, but also prove that specific behavior relevant in the work context (cooperation, contact) is a function of competence and warmth stereotypes and emotions elicited. In any case, future research developing measures of competitiveness, more appropriate for analyzing prejudice and discrimination in the working world, will be needed.

In spite of these limitations, our study contributes to the literature on gender segregation in the labor market by analyzing perceptions of status, competition, warmth and competence stereotypes, emotional reactions and intended behaviors as a function of occupational status and organizational context, two intersectional variables that exercise an important impact on gender inequalities and that are not usually jointly addressed. In addition, this research highlights the dual role played by emotions (admiration and envy) in confirming stereotypes and, in turn, motivating behaviors. As a result, men continue to outstrip women in the area of instrumental competence (Ridgeway, 2001) and also in the area of interpersonal competence, especially in low status positions.

## Ethics Statement

The study received approval from the UNED Ethics Committee and has been performed in accordance with the ethical standards of the Declaration of Helsinki. Participants in the final sample consented to participate in the study, and they could withdraw from the study whenever they wanted.

## Author Contributions

CG-A was involved in conception and design of the study, in the data collection, in the data analysis and manuscript writing, and read and approved the submitted version. IC and FM were involved in conception and design of the study, in manuscript writing and revisions, and read and approved the submitted version.

## Conflict of Interest Statement

The authors declare that the research was conducted in the absence of any commercial or financial relationships that could be construed as a potential conflict of interest.
